# Nutrition Status of Female Winter Sports Athletes

**DOI:** 10.3390/nu15204472

**Published:** 2023-10-22

**Authors:** María José Jiménez-Casquet, Javier Conde-Pipó, Ignacio Valenzuela-Barranco, Raquel Rienda-Contreras, Fátima Olea-Serrano, Cristina Bouzas, Josep A. Tur, Miguel Mariscal-Arcas

**Affiliations:** 1 Health Science and Nutrition Research (HSNR-CTS1118), Department of Nutrition and Food Science, School of Pharmacy, University of Granada, 18071 Granada, Spain; mariajosejcf@correo.ugr.es (M.J.J.-C.); javiercondepipo@gmail.com (J.C.-P.); folea@ugr.es (F.O.-S.); 2 Cetursa Sierra Nevada, Sierra Nevada-Monachil, 18196 Granada, Spain; ignacio.valenzuela@cetursa.es; 3 Department of Sports and Women, Andalusian Federation of Winter Sports (FADI), 18008 Granada, Spain; raquel.rienda@gmail.com; 4 Instituto de Investigación Biosanitaria de Granada (ibs.GRANADA), 18012 Granada, Spain; 5 Research Group on Community Nutrition and Oxidative Stress, University of Balearic Islands-IUNICS & IDISBA, 07122 Palma de Mallorca, Spain; cristina.bouzas@uib.es (C.B.); pep.tur@uib.es (J.A.T.); 6 Centro de Investigación Biomédica en Red Fisiopatología de la Obesidad y la Nutrición (CIBEROBN), Institute of Health Carlos III, 28029 Madrid, Spain; 7 Health Research Institute of the Balearic Islands, 07120 Palma de Mallorca, Spain

**Keywords:** female athletes, winter sports, nutrition, altitude, body composition

## Abstract

Eating disorders, especially restrictive eating, are common among female athletes. There are two main types of winter sports: those that are practiced outdoors on snow (−25 to +5 °C and 2500 m), such as alpine skiing and snowboarding, and those that are practiced indoors on ice (5–10 °C at low altitude), such as figure skating and ice hockey. The aim of this research was to identify the nutritional status and potential risk of female athletes practicing winter sports, considering the altitude of training. The sample was composed of 58 women (aged 19.81 years (SD: 12.61)) who were competitors in some winter sports. Anthropometrics and nutritional variables were taken. Statistically significant differences were found between HA and LA groups for all the characteristics except thigh skinfold, and neither group had an energy intake (EI) that matched their total energy expenditure (TEE). Both groups met at least two-thirds of the RDI for all minerals and vitamins except iodine, fluorine, vitamin D, vitamin E, and retinol. This study suggests that female winter sports athletes have insufficient energy, vitamin, and mineral intake, which can be worsened with altitude.

## 1. Introduction

Eating disorders, especially restrictive eating, are common among female athletes [1]. Frequently, they report low energy intake due to high training demands and a sports culture that is often focused on aesthetics from which winter sports are not exempt [2].

There are two main types of winter sports. On the one hand, those that are practiced outdoors on snow, such as alpine skiing or snowboarding, expose athletes to cold temperatures ranging from −25 to +5 °C and high-altitude conditions above 2500 m. On the other hand, those that are practiced indoors on ice, such as figure skating and ice hockey, also expose athletes to cold conditions, with average temperatures of 5–10 °C but at low altitudes [3,4]. Ice hockey is a contact team sport with intermittent bouts of high intensity [5,6], in which players are characterized by both muscle strength and endurance muscle power [2,7,8]. Thus, this sport requires aerobic and anaerobic metabolism as energy sources [9]. Figure skating requires aerobic and anaerobic endurance as well [10]. Currently, female athletes incorporate higher and more frequent jumps, spins, spiral elements, and steps that require relatively low body weights and good athleticism [10,11]. Alpine skiing and snowboarding, for their part, are considered intervallic strength-endurance sports performed at medium-high intensity [12].

These cold and altitude conditions, as well as the specific requirements of each sport, result in a combination of environmental stress and metabolic challenges that accelerate the onset of fatigue, decrease performance, and have several nutritional implications [3,13]. Moreover, most winter sports athletes undergo periods of highly intensive training, requiring increased energy and nutrient intakes, as well as adequate nutrition and hydration before, during, and after training [9].

Altitude exposure induces diuresis, reduces thirst, and increases ventilation in an environment of low humidity, resulting in a reduction in total body water and, with it, a possible state of dehydration [3,14]. In the case of alpine skiing, muscle damage induced by muscular ischemia, hypoxia, and increased utilization of glycogen can be minimized by maintaining hydration with carbohydrate–protein beverages [15]. Ice hockey, despite the cold and low-altitude environment, is a high-intensity sport that results in a high sweat rate and a subsequent loss of sodium and other electrolytes, particularly during match sessions [16]. Staying hydrated by ingesting a sports drink containing carbohydrates and electrolytes helps preserve performance while reducing thermal and perceptual strains [5,17,18]. Upon ascent to altitude, energy expenditure increases, and weight loss frequently occurs, averaging ~1.4 kg per week, which is explained by appetite suppression, the increase in energy requirements to maintain core temperature, and, at the same time, the use of protein as a metabolic fuel [3]. Therefore, one of the goals for winter sports athletes is to ensure that energy and fluid intakes are appropriate [19].

The adverse conditions in which winter sports are practiced condition the intake of micronutrients, mainly increasing the needs for vitamin D since it is a key regulator of calcium homeostasis and iron for its importance in oxygen transport and energy metabolism [19,20].

Despite the popularity of winter sports, there are no defined nutrition guidelines, and there are only a few reviews and research papers about this topic [19]. It is necessary to deepen both the requirements and the dietary habits of winter athletes to improve their health and optimize performance.

Nutritional deficiencies in female athletes cause numerous health problems, as well as a worsening of sports performance, because of these nutritional deficiencies. Awareness of these deficiencies and their prevention should be a major aspect for any sports manager [21,22].

Accordingly, the aim of this research was to identify the nutritional status and potential risk of female athletes practicing winter sports, considering the altitude of training.

## 2. Materials and Methods

The study design was cross-sectional, descriptive, and comparative. The Andalusian Federation of Winter Sports (Spain) collaborated on the project with the Department of Nutrition and Food Science of the University of Granada (Spain). The study protocols and procedures were developed in accordance with the standards of the Declaration of Helsinki and approved by the Research Ethics Committee of the University of Granada, Spain (ref. 1162/CEIH/2020). Prior to participating in the study, all participants were informed of the objectives of the research, and they provided their written informed consent.

The sample was composed of 58 women (aged 19.81 years (SD: 12.61)) who were competitors in some winter sports. The sports practiced were alpine skiing (43.10%), freestyle skiing or snowboarding (6.90%), ice hockey (25.86%), and figure skating (24.14%). Subsequently, the sample was divided into two groups according to the sport practiced: high altitude (HA), including alpine skiing, freestyle skiing, and snowboarding, and low altitude (LA), including figure skating and ice hockey. The skiers of this sample usually train in Sierra Nevada Ski Resort (Granada, Spain), where the average altitude is 2800 m with a maximum altitude of 3300 m, while the ice sports sample trains in Granada city, at a constant altitude of 738 m.

The inclusion criteria were being female, being a minimum of 10 years of age, and competing in the Andalusian Winter Sports Federation during the study. Players who were injured or ill during the study were excluded. Data were collected during the competition season. All data were collected during the winter period, between January and February.

Anthropometric variables were taken by trained regular personnel of the European Leagues, certified by the ISAK, International Society for the Advancement of Kinanthropometry, with a technical measurement error of 0.04% for basic measurements and 2.12% for skinfolds, following the international standards recommended by the ISAK. All measures were taken in the morning. Height was measured in centimeters using a wall-mounted stadiometer (Seca 214; SECA Deutschland, Hamburg, Germany), and weight was measured in kilograms with a high-precision scale (Tanita BC-418; Tanita, Tokyo, Japan). On the measurement days, athletes should neither have performed high-intensity exercise the previous day nor have performed training or stretching sessions on the same day. All participants were weighed wearing light clothing and barefoot (0.6 kg was subtracted from the total for clothing) [23]. Body mass index (BMI) was calculated by dividing the weight in kilograms by the square of height in meters (kg/m^2^). The skinfolds were measured with a Holtain plicometer (Crosswell, Crymych, Pembs, Wales, UK). The skinfolds measured were bicipital, tricipital, subscapular, supra-iliac, abdominal, mid-leg, and thigh. Faulkner’s fat estimation formula was used [24]. All anthropometric measurements were taken in triplicate, using the mean of both for subsequent analysis.

Basal metabolic rate (BMR) was estimated through the Harris–Benedict formula [25]. Total energy expenditure (TEE) was estimated following the formula proposed by FAO, WHO, and United Nations University [26,27,28,29] based on BMR. Depending on the volume and training load per week that each athlete presented, a physical activity level (PAL) factor of 1.56, 1.64, or 1.82, specific for women, was applied for the final estimate.

The nutritional method was a 3-day 24 h recall questionnaire (R24h), previously validated by the research group [30], which contains the diet followed by the subjects in the last days and allows estimating the intake of food, energy, and essential nutrients. It was completed through face-to-face individual interviews by specifically trained interviewers. The third questionnaire covered three days and 24 h/day for three non-consecutive days, including one non-working day [31,32,33,34,35]. Subsequently, essential micronutrient intakes were compared to their corresponding recommended dietary intake (RDI) estimated for the general Spanish female population [28,29], given that there are no specific recommendations for the Spanish population practicing winter sports or for athletes in general. With these micronutrient recommendations, in which there are differences regarding age, the average RDI was calculated. Questionnaires were treated with cut-off points as unreliable following the criteria established by Goldberg et al. [31,32,33,34,35], taking into account the EI results, daily energy expenditure (from 3 × 24 h recall), and BMR [26,27].

### Statistics

Nutritional intakes were estimated using the Dial program (Copyright © 2015 Alce Ingeniería) in combination with the AUSNUT 2011-13 food nutrient database [36,37]. Statistical analysis was performed with the statistical computing software R (v 4.1.2.; R Core Team, Vienna, Austria). The normality of the variables was analyzed using the Kolmogorov–Smirnov test with the Lillieforts correction, and homoscedasticity was analyzed with the Levene test. Means and standard deviations were used for basic descriptions. For the comparisons between groups of continuous variables, the nonparametric Mann–Whitney U test was used, and to calculate the effect size, we used the Cohen’s d index. In the case of bivariate correlations, Spearman’s rho correlation coefficient was used. All reported *p* values were based on the two-tailed test, and the level of statistical significance for all tests was set at 95%.

## 3. Results

Table 1 shows a comparison of the anthropometric characteristics by altitude group. Statistically significant differences were found between HA and LA groups for all the characteristics except thigh skinfold (*p* = 0.473; η^2^ = −0.15; CI = −0.68–0.38), with the highest means corresponding to the high-altitude (HA) group.

**Figure 1:**
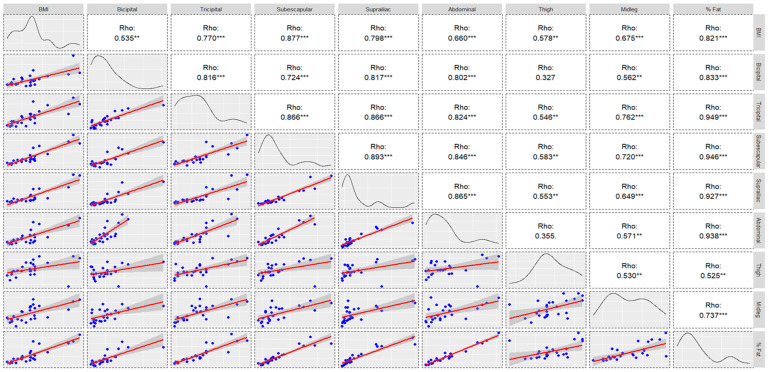
Chart of correlations, density curve, and regression models of anthropometric measures for high-altitude group (** *p* ≤ 0.010; *** *p* ≤ 0.001).

**Figure 2:**
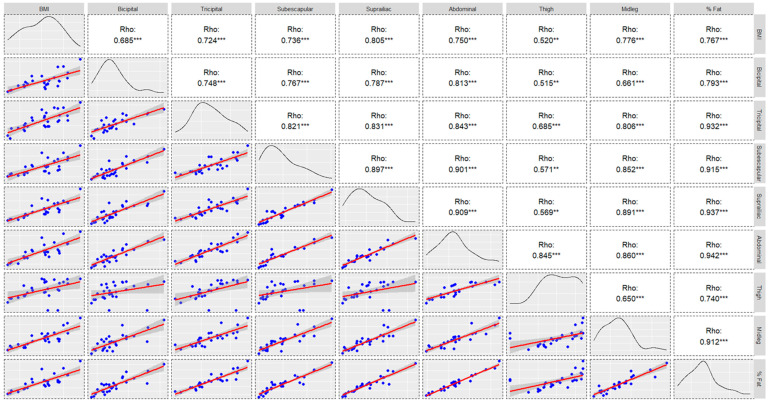
Chart of correlations, density curve, and regression models of anthropometric measures for low-altitude group (** *p* ≤ 0.010; *** *p* ≤ 0.001).

Bivariate correlations between these characteristics are shown in Figure 1 and Figure 2. For the percentage of fat in both groups, the highest correlation values were found in the tricipital, suprascapular, supra-iliac, and abdominal folds (r > 0.9).

Regarding macronutrient intake, as shown in Table 2, neither group had an energy intake (EI) that matched their total energy expenditure (TEE). The HA group had the highest mean intakes of water (*p* = 0.035; η^2^ = −0.52; CI = −1.04–0.01), carbohydrates (*p* = 0.018; η^2^ = 0.54; CI = 0.11–1.16), soluble fiber (*p* = 0.009; η^2^ = 0.54; CI = 0.02–1.06), and indissoluble fiber (*p* = 0.008; η^2^ = 0.63; CI = 0.10–1.16). No statistically significant differences were found between the HA and LA groups with respect to the caloric profile (*p* ≤ 0.005), but with respect to the lipid profile, SFA was higher in the LA group (*p* = 0.001; η^2^ = 1.22; CI = 0.65–1.78).

**Figure 3:**
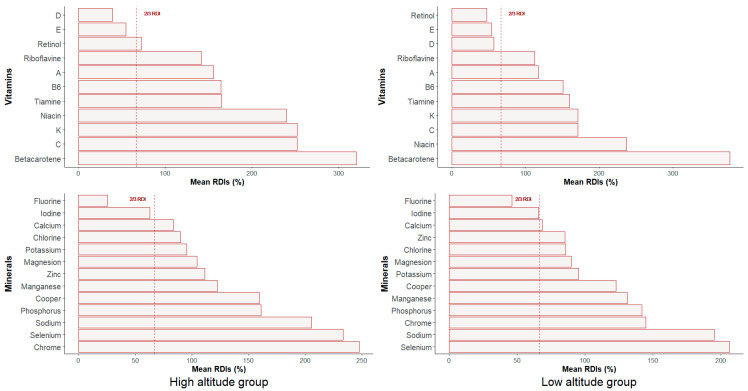
Percentage of adjustment to the RDIs of minerals and vitamins intakes by altitude group.

Micronutrient intake means were compared between groups and against recommended dietary intakes (RDIs) for the general Spanish female population accordingly (Table 3 and Table 4, Figure 3).

No statistically significant differences were found between HA and LA groups in mineral intake (*p* ≥ 0.05). The percentage of calcium intake respecting RDI was higher in the HA group (*p* = 0.059; η^2^ = 0.38; IC = −0.17–0.92). Both groups met at least two-thirds of the RDI for all minerals except iodine and fluorine intake.

With respect to vitamin intake, only retinol showed statistically significant differences between the HA and LA groups (*p* = 0.025; η^2^ = 0.48; IC = −0.07–1.02), with higher values in the HA group. The percentage of riboflavin intake with respect to RDI was higher in the HA group (*p* = 0.054; η^2^ = 0.62; IC = 0.06–0.92).

Both groups met at least two-thirds of the RDI for all vitamins except vitamins D and E and retinol in the LA group and vitamins D and E in the HA group.

## 4. Discussion

The main findings from this study were that both the HA group and LA group had similar and adequate dietary patterns with respect to macronutrients and micronutrients but insufficient energy intake, which corroborates what was published by Vazquez Franco et al. in 2020 on the female athlete triad because of energy intake deficiencies where they recommended for Spanish women a macronutrient calorie profile of more than 55% of energy in the form of CH, 10–15% of energy in the form of protein, and between 30–35% of energy in the form of lipids [21]. Differences were found to be statistically significant between the HA and LA groups in the total amount of water, carbohydrate, fiber, and retinol intake, with the higher values in the HA group excepting for retinol.

The micronutrient intakes are in line with the estimated RDIs for the general Spanish female population, in which both groups had a low to two-thirds intake of iodine, fluorine, vitamin D, vitamin E, and, just in LA group, retinol intakes. These results may be due to the high prevalence of the Mediterranean diet pattern in all southern regions of Spain [24]. Nevertheless, considering the sample sex, type of sport practiced, and altitude, some of these differences can be important. What must also be taken into account is that the nutritional energy needs of female athletes compared to the general population are greater, although there are currently no recommendations for the Spanish population practicing winter sports against which to compare intake.

### 4.1. Anthropometry

The percentage of body fat (BF) in the LA group was like that in previous studies focusing on women, reporting values of 17.2–17.6% in figure skating [10,11] and 13–18.9% in ice hockey [38,39]. However, in the HA group, BF was lower than those reported for alpine skiers in several reviews of the literature (18.4–22.1%) [40,41]. This may be because most of the studies focusing on skiers used samples from North/Central European regions or North America, places where skiing is popular [42] but where eating habits are more Westernized and unhealthier [43]. Differences between groups can be explained first by the metabolic response to the altitude of women, relying on fat as fuel to a greater extent during submaximal exercise and at rest [3], and second, by the improved body composition associated with improvements in insulin sensitivity found in alpine skiers [44,45].

### 4.2. Macronutrients

In the current study, in both groups, EI was not enough to cover the estimated TEE, with the greatest difference in the HA group. Therefore, the athletes were probably at risk of low energy availability (LEA), a dangerous state that should be monitored by both parents and coaches. LEA occurs when there is insufficient energy to maintain basic physiological and endocrine functions, causing female athlete’s triad, reduced performance, chronic fatigue sensation, low mood, poor concentration, impaired immune system, absence of menarche, and poor bone health [19,21,46]. In addition, LEA is associated with eating disorders [1]. Recent studies placed the LEA threshold for healthy female athletes at an intake lower than or equal to 30 kcal/kg FFM/day [47].

Fluid balance plays a crucial role in exercise performance, as well as in winter sports [14]. Ice hockey players became mildly dehydrated by 1.3–4.3% body mass [17]. In athletes training at altitude, it is suggested to have a fluid intake of 4.5 L per day and rehydration at 1.5 L of fluid per kg of body mass lost after training [3,19]. The fluid intake in the HA group was far from that suggested by these authors. One of the reasons is the lack of on-snow availability, and because of that, skiers should make a conscientious effort to stop skiing to go into the lodge and get their drinks [14]. Another reason is the necessity of holistic sports nutrition education [19].

In intermittent sports, such as alpine skiing or ice hockey, where strength and power are predominant, greater concentrations of glycogen are depleted, of which an intake of carbohydrates of 5–10 g·kg^−1^·day^−1^ is adequate [25], figures that are very distant of those consumed by both groups studied. Also, in the LA group, the caloric profile differed from that reported in other studies with professional ice hockey players, with a significantly lower proportion of energy from carbohydrates (18–32%) and a higher proportion of energy from protein (38%) [8], which could affect physical performance [5,8] since during an ice hockey match, muscle glycogen has been reported to decline between 38–88% [8]. It could even affect the growth of those athletes who have not yet completed their own growth because of energy/protein imbalance. Nevertheless, in the HA group, the results differ in percentages of energy from total fat and SFA, being both considerably higher compared with WHO dietary guidelines for the European Region [48] and recommendations established previously by Butterfield [4] for high-altitude sports (30% and 10%, respectively). This higher energy intake from total fat and SFA is not reflected in higher body fat and may be due to the different behaviors of fat metabolism at different altitudes, as discussed in the previous section.

If we also consider the higher intake of fiber and fructose, mainly present in fruits and vegetables, everything seems to indicate that the dietary habits of the LA group are healthier than those of the HA group.

### 4.3. Micronutrients: Vitamins

Iron, calcium, and vitamin D are micronutrients that female athletes commonly consume in low quantities [2]. The high prevalence of vitamin D deficiency is a worldwide problem, affecting athletes in a wide range of sports, especially in the winter months [49]. Given the impact of vitamin D on health, particularly bone health [50], and the high risk of bone injury from falls or impacts in all sports studied, athletes, particularly those playing indoor sports, should consider ways to increase vitamin D status through diet, supplements, and sun exposure [20]. In our study, no group met two-thirds of RDI for vitamin D intake, although the HA group might have compensated for the low vitamin D intake with higher exposure to ultraviolet B (UVB) sunlight [49]. These results are consistent with other studies conducted in alpine skiers [51], hockey players [2,52], and other team sports with Spanish elite athletes [49].

Free radical production during exercise is increased by environmental factors, such as altitude, low temperatures, and increased UV light exposure [53,54]. Hence, it is crucial for all athletes, with a particular emphasis on the HA group, to ensure they meet at least two-thirds of the recommended daily intake (RDI) for antioxidants, including vitamins A, C, and E; retinol; and betacarotene. Nevertheless, both groups exhibited a deficiency in vitamin E, likely attributed to the inadequate consumption of vegetables prevalent in the Westernized diet adopted by Mediterranean youth in recent times [43].

### 4.4. Micronutrients: Minerals

For female athletes engaged in winter sports, calcium and iron are minerals that warrant particular attention because of their respective functions in maintaining bone health and supporting erythrocyte production [3,50]. Nevertheless, unlike other similar studies that showed a low intake of potassium, calcium, or iron [2,19], only the intake of fluorine and iodine showed deficiencies, with possible consequences for bone structure and thyroid disease, which are common in female athletes. These conditions may be exacerbated by strenuous exercise but are easily corrected by increasing the intake of tea, seafood, dairy products, and/or iodized salt [54,55].

### 4.5. Menstrual Cycle

It is necessary to consider that female athletes may experience heightened nutritional requirements during the menstrual cycle because of fluctuating hormonal levels. The luteal phase, distinguished by elevated estrogen and progesterone levels, is associated with increased protein oxidation, requiring more proteins, concretely lysine, than during the follicular phase. Nevertheless, the follicular phase is when carbohydrate oxidation rates are elevated and when carbohydrate feeding is more important [56]. Furthermore, female athletes have an increased need for zinc in the mid-luteal phase and for folate, riboflavin, and B12 in the follicular phase [56].

### 4.6. Strengths and Limitations

The main strengths of the current study were that the subjects were female and that both the HA and LA groups had similar dietary patterns in terms of macronutrients and micronutrients but insufficient energy intake (EI). The current study also has limitations. The main limitations are its descriptive cross-sectional design and the sample size used, which do not allow for the establishment of any causal inferences. These results need to be interpreted with caution. Firstly, dietary habits were reported by the participants, and previous studies with female athletes have documented the under-reporting of dietary intake when self-reporting [2]. Secondly, the current study relies on several assumptions when predicting average daily energy expenditure. To enhance the precision of these estimates, it would be convenient to employ more accurate measures, such as doubly labeled water, heart rate monitoring, or accelerometers. Additionally, the RDIs for micronutrients used in this study are estimates for the general Spanish female population, and the adjustment percentage is based on the average RDI across the age range of these athletes. This approach may lead to the potential underestimation of certain RDIs, and therefore, specific RDIs tailored for winter sports athletes, considering the altitude factors are warranted. Finally, given that these athletes become professional at an earlier age, these RDIs should be established considering different age ranges, with a specific focus on distinguishing between adolescents and adults.

It should also be noted that, to date, studies of nutrition in winter sports specific to women or that incorporate women in their samples are scarce and, therefore, necessary.

## 5. Conclusions

This study suggests that female winter sports athletes exhibit inadequate energy intake relative to their estimated energy expenditure, coupled with deficiencies in the intake of vitamins (D and E) and minerals (fluoride and iodine). These deficiencies can be exacerbated by exposure to high altitudes or during the menstrual cycle. It is crucial to acknowledge that such nutritional inadequacies may negatively impact both the performance and health of athletes. Therefore, addressing these nutritional concerns in their preparation and athletic performance becomes imperative, considering altitude-specific factors and the specific nutritional requirements of women.

## Figures and Tables

**Table 1:** Anthropometric characteristics of the study sample by altitude group.

Variable (Mean, SD)	Sample (N = 58)	Group		Sig.	Effect Size	
		High Altitude HA (n = 29)	Low Altitude LA (n = 29)	p	d	IC
Age (years)	19.81(12.61)	21.55(15.47)	18.07(8.84)	0.522	0.21	(−0.31, 0.72)
Height (cm)	153.16(17.51)	149.17(21.23)	157.14(11.85)	0.230	−0.46	(−0.98, 0.06)
Weight (kg)	51.70(17.82)	46.21(16.70)	57.19(17.46)	0.031	0.64	(0.11, −1.17)
BMI (kg/m^2^)	21.28(4.50)	19.98(4.38)	22.59(4.31)	0.028	0.60	(0.07, 1.12)
Tricipital skinfold (mm)	16.46(6.37)	14.58(6.25)	18.15(6.08)	0.021	0.58	(0.04, 1.12)
Bicipital skinfold (mm)	9.23(5.01)	7.64(4.12)	10.65(5.36)	0.010	0.62	(0.08, 1.16)
Subscapular skinfold (mm)	12.77(7.47)	10.61(6.80)	14.71(7.63)	0.012	0.57	(0.02, 1.10)
Supra-iliac skinfold (mm)	13.01(8.68)	10.42(8.42)	15.33(8.37)	0.008	0.59	(0.04, 1.12)
Abdominal skinfold (mm)	16.16(9.04)	13.84(8.66)	18.31(9.01)	0.033	−0.51	(−1.06, 0.05)
Thigh skinfold (mm)	24.24(10.01)	23.45(9.00)	24.94(10.95)	0.473	−0.15	(−0.68, 0.38)
Midleg skinfold (mm)	16.89(7.42)	14.15(5.31)	19.34(8.23)	0.009	0.74	(0.19, 1.29)
Faulkner body fat (%)	14.59(4.38)	13.27(4.05)	15.77(4.39)	0.013	0.59	(0.05, 1.13)

**Table 2:** Macronutrients daily intake by altitude group.

Variable	Sample	Group		Sig.	Effect Size	
		High Altitude HA	Low Altitude LA	p	d	IC
BMR, kcal *	1272.63(238.86)	1197.33(261.85)	1347.93(189.30)	0.102	0.66	(0.13, 1.19)
TEE, kcal *	2068.52(613.89)	2115.16(825.86)	2021.89(283.94)	0.699	0.15	(−0.37, 0.67)
Energy intake, kcal *	1755.76(453.28)	1652.86(409.72)	1858.66(477.98)	0.125	−0.46	(−0.98, 0.60)
Water, g *	2471.48(910.90)	2242.00(861.03)	2700.97(915.78)	0.035	−0.52	(−1.04, 0.01)
Protein, g *	80.81(28.30)	76.11(19.88)	85.51(34.48)	0.262	−0.33	(−0.85, 0.19)
Carbohydrate, g *	165.06(47.59)	150.51(46.54)	179.62(44.78)	0.018	0.64	(0.11, 1.16)
Lipid, g *	80.21(30.33)	77.65(28.82)	82.76(32.07)	0.797	−0.17	(−0.68, 0.35)
*Simple carbohydrate*						
Glucose, g *	7.78(4.59)	6.25(3.93)	9.32(4.75)	0.004	0.70	(0.17, 1.23)
Fructose, g *	9.73(6.01)	8.11(5.90)	11.35(5.77)	0.013	0.56	(0.03, 1.08)
Lactose, g *	9.12(6.48)	7.69(5.12)	10.56(7.41)	0.135	−0.45	(−0.97, 0.07)
Soluble fiber, g *	3.61(2.89)	2.85(1.43)	4.37(3.70)	0.009	0.54	(0.02, 1.06)
Indissoluble fiber, g *	6.34(4.50)	4.97(2.65)	7.71(5.51)	0.008	0.63	(0.10, 1.16)
Cholesterol, mg *	352.57(21.99)	343.07(218.70)	363.20(208.17)	0.662	−0.09	(−0.63, 0.45)
*Caloric profile*						
Proteins, %	18.47(4.46)	18.80(4.32)	18.15(4.65)	0.888	0.15	(−0.37, 0.66)
Carbohydrates, %	38.07(7.91)	36.71(8.19)	39.43(7.52)	0.269	−0.35	(−0.86, 0.17)
Lipids, %	40.49(7.20)	41.62(7.28)	39.36(7.06)	0.297	031	(−0.21, 0.83)
*Lipids profile*						
SFA, %	11.41(4.23)	13.62(2.99)	9.20(4.17)	0.001	1.22	(0.65, 1.78)
MUFA, %	15.84(5.93)	16.63(6.35)	15.04(5.49)	0.291	0.27	(−0.25, 0.78)
PUFA, %	5.08(2.57)	4.99(2.49)	5.16(2.70)	0.779	−0.06	(−0.58, 0.45)

Note: * means (SD). BMR: basal metabolic rate; TEE: total energy expenditure; SFA: saturated fatty acids; MUFA: monounsaturated fatty acids; PUFA: polyunsaturated fatty acids.

**Table 3:** Mineral intake and adjustment percentage to recommended dietary intake by altitude group.

Mineral		Sample	Group		Sig.	Effect Size	
			High Altitude HA	Low Altitude LA	p	d	IC
Calcium	Intake, mg	815(463.13)	855.88(349.29)	778.50(549.28)	0.123	0.17	(−0.38, 0.71)
	% RDI	75.70(40.81)	83.75(36.39)	68.52(43.77)	0.059	0.38	−0.17, 0.92)
Iron	Intake, mg	14.55(6.38)	15.09(7.77)	14.07(4.94)	0.694	0.16	(−0.38, 0.70)
	% RDI	110.48(59.42)	117.26(59.32)	104.43(59.94)	0.322	0.22	(−0.33, 0.75)
Iodine	Intake, µg	82.72(35.10)	81.58(34.19)	83.74(36.48)	0.992	−0.06	(−0.60, 0.48)
	% RDI	64.50(30.31)	62.82(29.09)	65.99(31.81)	0.823	−0.10	(−0.64, 0.44)
Zinc	Intake, mg	9.73(2.99)	9.78(3.00)	9.69(3.049	0.936	0.03	(−0.51, 0.57)
	% RDI	97.68(49.56)	111.37(56.34)	85.45(39.76)	0.085	0.54	(−0.01, 1.08)
Magnesium	Intake, mg	285.32(109.85)	292.20(125.18)	279.18(96.06)	0.796	0.12	(−0.42, 0.66)
	% RDI	96.69(43.64)	104.39(49.37)	89.81(37.88)	0.314	0.34	(−0.21, 0.88)
Potassium	Intake, mg	2944.21(987.14)	3004.68(1204.81)	2890.21(761.59)	0.852	0.12	(−0.43, 0.65)
	% RDI	95.39(37.59)	101.28(43.96)	90.13(30.69)	0.518	0.30	(−0.25, 0.84)
Sodium	Intake, mg	2917.57)	2962.88(1524.79)	2877.11(1589.22)	0.839	0.06	(−0.48, 0.59)
	% RDI	200.03(109.41)	205.63(110.84)	195.03(109.90)	0.730	0.10	(−0.44, 0.64)
Selenium	Intake, µg	96.18(46.75)	93.20(47.86)	98.83(46.45)	0.796	−0.12	(−0.66, 0.42)
	% RDI	219.15(128.30)	233.32(154.83)	206.49(100.21)	0.734	0.21	(−0.33, 0.75)
Manganese	Intake, mg	2.88(1.47)	2.58(1.31)	3.15(1.57)	0.151	−0.39	(−0.93, 0.16)
	% RDI	127.22(58.34)	122.62(49.48)	131.33(65.89)	0.755	−0.15	(−0.69, 0.39)
Cooper	Intake, mg	1.52(0.78)	1.63(1.05)	1.41(0.43)	0.844	0.28	(−0.26, 0.82)
	% RDI	140.18(79.08)	159.43(103.30)	122.99(43.70)	0.322	0.47	(−0.08, 1.01)
Chrome	Intake, µg	48.58(47.68)	59.60(44.88)	38.75(20.58)	0.127	0.44	(−0.10, 0.99)
	% RDI	193.36(206.89)	247.56(274.49)	144.96(101.55)	0.128	0.47	(−0.08, 1.01)
Chlorine	Intake, mg	1981.66(792.22)	2019.80(919.47)	1947.61(674.31)	0.907	0.09	(−0.45, 0.63)
	% RDI	87.94(36.21)	90.07(41.67)	86.03(31.20)	0.852	0.11	(−0.43, 0.65)
Fluorine	Intake, mg	2.34(1.06)	2.22(1.12)	2.44(1.01)	0.446	−0.20	(−0.74, 0.33)
	% RDI	36.59(37.45)	25.64(29.58)	46.36(41.39)	0.123	0.57	(0.02, 1.12)
Phosphorus	Intake, mg	1324.98(396.61)	1347.28(410.20)	1305.07(390.53)	0.957	0.11	(−0.43, 0.64)
	% RDI	150.92(65.08)	161.07(61.87)	141.85(67.63)	0.210	0.30	(−0.25, 0.84)

Note: Data are presented as means (SD). % RDI: adjustment percentage of recommended dietary intake for the general Spanish female population.

**Table 4:** Vitamins daily intake and adjustment percentage to recommended dietary intake by altitude group.

Vitamin		Sample	Group		Sig.	Effect Size	
			High Altitude HA	Low Altitude LA	p	d	IC
Thiamine	Intake, mg	1.59(0.69)	1.54(0.72)	1.78(0.73)	0.865	0.07	(−0.47, 0.61)
	% RDI	162.31(73.20)	164.98(73.44)	159.92(74.26)	0.809	0.07	(−0.47, 0.61)
Riboflavin	Intake, mg	1.69(0.60)	1.84(0.66)	1.55(0.51)	0.107	0.50	(−0.05, 1.05)
	% RDI	126.37(49.54)	141.88(58.52)	112.52(35.52)	0.054	0.62	(0.06, 1.05)
Niacin	Intake, mg	35.03(10.85)	34.92(11.56)	35.15(10.24)	0.809	−0.02	(−0.56, 0.52)
	% RDI	238.53(77.73)	242.30(86.95)	234.31(67.44)	0.914	0.10	(−0.44, 0.64)
B6	Intake, mg	2.19(0.80)	2.12(0.68)	2.25(0.90)	0.661	−0.15	(−0.69, 0.39)
	% RDI	157.34(62.08)	164.35(58.52)	151.07(65.52)	0.309	0.21	(−0.33, 0.75)
Folic acid	Intake, µg	275.75(132.40)	278.60(146.28)	273.21(121.35)	0.950	0.04	(−0.50, 0.58)
	% RDI	88.74(46.67)	96.19(50.27)	82.10(43.03)	0.170	0.30	(−0.24, 0.84)
B12	Intake, µg	4.77(4.109	4.42(2.17)	5.08(5.28)	0.590	−0.16	(−0.70, 0.38)
	% RDI	174.39(199.31)	141.07(77.88)	204.15(263.00)	0.475	−0.32	(−0.86, 0.23)
Vitamin C	Intake, mg	118.32(94.48)	121.60(116.33)	115.39(71.73)	0.816	0.07	(−0.47, 0.60)
	% RDI	210.00(185.74)	252.30(245.17)	170.84(94.22)	0.489	0.45	(−0.11, 0.99)
Vitamin A	Intake, µg	901.00(503.10)	988.84(608.11)	822.57(381.11)	0.636	0.33	(−0.21, 0.87)
	% RDI	135.62(93.56)	155.48(105.29)	117.23(78.78)	0.785	−0.04	(−0.58, 0.51)
Vitamin D	Intake, µg	2.57(2.40)	1.98(1.55)	3.09(2.89)	0.172	−0.47	(−1.02, 0.08)
	% RDI	48.72(40.61)	39.59(30.92)	57.18(46.89)	0.226	−0.44	(−0.99, 0.11)
Vitamin E	Intake, mg	8.15(3.75)	8.19(4.59)	8.10(2.89)	0.655	0.02	(−0.52, 0.56)
	% RDI	54.30(25.00)	54.61(30.58)	54.02(19.29)	0.655	0.02	(−0.52, 0.56)
Vitamin K	Intake, µg	168.43(144.84)	179.08(172.28)	158.92(117.56)	0.907	0.14	(−0.40, 0.68)
	% RDI	179.01(169.36)	210.36(197.86)	151.02(136.81)	0.462	0.35	(−0.19, 0.89)
Retinol	Intake, µg	384.58(343.87)	469.68(388.07)	308.63(284.94)	0.025	0.48	(−0.07, 1.02)
	% RDI	59.17(52.90)	72.25(59.70)	47.48(43.84)	0.462	0.35	(−0.19, 0.89)
Betacarotene	Intake, µg	2452.37(896.53)	2239.59(1658.70)	2690.70(2141.49)	0.538	−0.24	(−0.78, 0.31)
	% RDI	350.34(270.93)	319.94(236.96)	384.39(305.93)	0.538	−0.24	(−0.78, 0.31)

Note: Data are presented as means (SD). % RDI: adjustment percentage of recommended dietary intake for the general Spanish female population.

## Data Availability

There are restrictions on the availability of data for this trial due to the signed consent agreements around data sharing, which only allow access to external researchers for studies following the project’s purposes. Requestors wishing to access the trial data used in this study can make a request to mariscal@ugr.es.
